# Microarray comparative genomic hybridisation analysis of intraocular uveal melanomas identifies distinctive imbalances associated with loss of chromosome 3

**DOI:** 10.1038/sj.bjc.6602834

**Published:** 2005-10-25

**Authors:** S Hughes, B E Damato, I Giddings, P S Hiscott, J Humphreys, R S Houlston

**Affiliations:** 1 Section of Cancer Genetics, Institute of Cancer Research, Sutton, SM2 5NG, UK; 2 Liverpool Ocular Oncology Center, Royal Liverpool University Hospital, Liverpool L7 8XP, UK; 3 Section of Molecular Carcinogenesis, Institute of Cancer Research, Sutton SM2 5NG, UK

**Keywords:** uveal melanoma, array CGH, regions of imbalance

## Abstract

Defining regions of genomic imbalance can identify genes involved in tumour development. Conventional cytogenetics has identified several nonrandom copy number alterations (CNA) in uveal melanomas (UVM), which include monosomy 3, chromosome 6 abnormalities and gain of 8q. To gain further insight into the CNAs and define the regions involved more precisely we analysed 18 primary UVMs using 1 Mb BAC microarray comparative genomic hybridisation (CGH). Our analysis showed that the most common genomic imbalances were 8q gain (78%), 6p gain (67%) and monosomy 3 (56%). Two distinct CGH profiles could be delineated on the basis of the chromosome 3 status. The most common genetic changes in monosomy 3 tumours, in our study, were gain of 8q11.21–q24.3, 6p25.1–p21.2, 21q21.2–q21.3 and 21q22.13–q22.3 and loss of 1p36.33–p34.3, 1p31.1–p21.2, 6q16.2–q25.3 and 8p23.3–p11.23. In contrast, disomy 3 tumours showed recurrent gains of only 6p25.3–p22.3 and 8q23.2–q24.3. Our approach allowed definition of the smallest overlapping regions of imbalance, which may be important in the development of UVM.

Uveal melanomas (UVM) are the most common primary intraocular malignant tumours (Stang *et al*, 2005). Despite successful treatment of the primary tumour approximately 50% of patients develop metastatic disease, which is usually unresponsive to chemotherapy and invariably fatal (Bergman *et al*, 2003). Tumours most commonly arise in the choroids with less than 10% developing in the iris and ciliary body. UVMs are classified as spindle, epithelioid, or mixed according to their histological appearance (McLean *et al*, 2004).

Cytogenetic analyses and allelic imbalance studies have identified a number of recurrent chromosomal aberrations in UVM, including loss of chromosome 3, and copy number alterations (CNA) on chromosomes 6 and 8 (Tschentscher *et al*, 2000; Aalto *et al*, 2001; Naus *et al*, 2001).

In this study we have, for the first time sought to further refine the regions of chromosomal imbalance in UVM by analysing tumours using BAC microarray CGH. The resolution of arrays in identifying regions of chromosome imbalance is dependent upon the number and distribution of the clones. The arrays used in this study were based on a previously reported BAC clone set (Fiegler *et al*, 2003) and we validated the detection of CNAs in a series of sex mismatch experiments through which incorrectly annotated clones were identified and excluded from subsequent analyses. The median coverage across the genome for the 3421 BACs included on the array was one clone every 1 Mb (range 500 Kb–4.5 Mb, excluding BACs spanning centromeres). Detection of copy number changes is influenced by factors including tumour heterogeneity and contamination with infiltrating lymphocytes. In this study, we restricted our analysis to tumours that have been verified to contain less than 10% normal cell contamination. This approach has allowed for the definition of chromosomal regions that represent the smallest overlapping regions of imbalance (SORI), which are likely to harbour oncogenes or tumour suppressor genes.

## MATERIALS AND METHODS

### Patient samples

The tumour samples used in this study (Table 1) were obtained from different patients attending the Liverpool Ocular Oncology Centre between 1994 and 1997. All tumours were primary lesions and the diagnosis of UVM was histologically confirmed in all cases. In the nine patients alive on the 15 August 2005, the follow-up had a median of 6.97 years, exceeding 1 and 5 years in 15 patients and 10 patients, respectively. Samples were obtained with informed consent and Local Ethical Review Board approval in accordance with the tenets of the Declaration of Helsinki.

### Array CGH

DNA was extracted from tumours using the QIAamp DNA Micro Kit (Qiagen, UK) and the DNA concentration was determined using the RediPlate 96 PicoGreen dsDNA Quantitation Kit (Invitrogen, UK). Both procedures were performed following manufacturers instructions. The genomic DNA arrays used in these experiments were obtained from the Cancer Research UK DNA Microarray Facility and consist of 3421 BAC and PAC clones, which provide an average genomic resolution of 1 Mb. Reference DNA (Promega, UK) and test DNA were labelled separately with Cy3 and Cy5 dyes (Amersham, UK) using the BioPrime Labelling Kit (Invitrogen, UK), following manufacturers instructions and as described previously (Douglas *et al*, 2004).

Test and reference DNAs (50 *μ*l each) were combined and precipitated together with 100 *μ*g of human Cot1 DNA (1 mg ml^−1^; Invitrogen, UK) and 50 *μ*l yeast tRNA (5 mg ml; Invitrogen, UK). The DNA pellets were resuspended in 10 *μ*l of sterile water prior to being mixed with 10 *μ*l of microarray hybridisation solution (Amersham, UK) and 20 *μ*l of deionised formamide (Sigma, UK). The reconstituted probes were then incubated at 72°C for 15 min followed by 30 min at 37°C. The probes were hybridised to BAC arrays and incubated for 48–72 h at 37°C in a humidified chamber. Slides were washed for 15 min at 42°C in 2 × SSC, 0.1% SDS, 15 min at 42°C in 50% formamide/2 × SSC, 30 min at 42°C in 2 × SSC, 0.1% SDS and 15 min at room temperature in 0.2 × SSC, before being dried by spinning in a centrifuge for 5 min at 150 ***g***.

### Data collection and analysis

The slides were scanned using an Axon GenePix 4000A confocal scanner, each fluorescence signal was collected separately and quantified with the GenePix Pro 3.0 software (Axon Instruments, USA). Spots were defined by use of the automatic grid feature of the software and manually adjusted where necessary. The data was normalised and analysed using Normalise Suite v2.4 (Beheshti *et al*, 2003), regions of loss or gain were determined as those that were 2 s.d. above the mean baseline for each separate sample.

## RESULTS AND DISCUSSION

**Figure 1:**
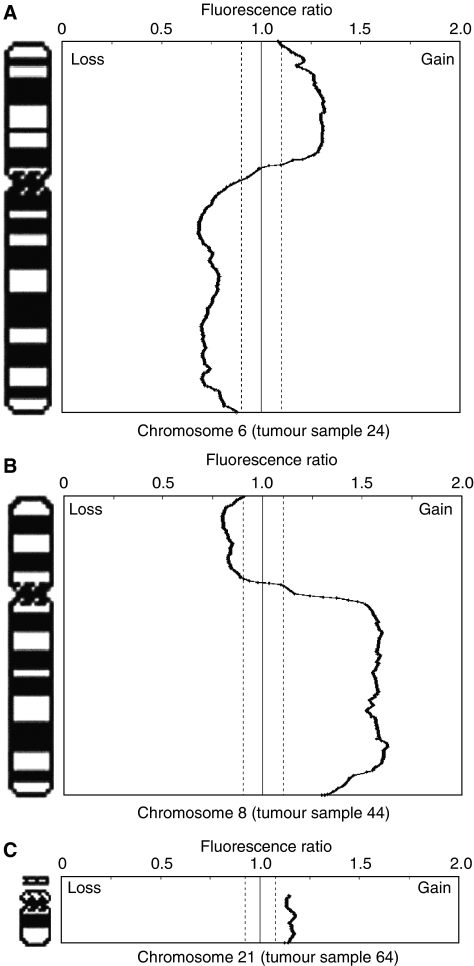
Examples of array CGH data, (**A**) 6p gain and 6q loss (tumour sample 24), (**B**) 8p loss and 8q gain (tumour sample 44) and (**C**) gain of 21 (tumour sample 64). The data was normalised and analysed using Normalise Suite v2.4 (Beheshti *et al*, 2003), regions of loss (left of the central line) or gain (right of the central line) were determined as those that were 2 s.d. (denoted by dashed black or gray lines) from the mean baseline for each separate sample.

**Figure 2:**
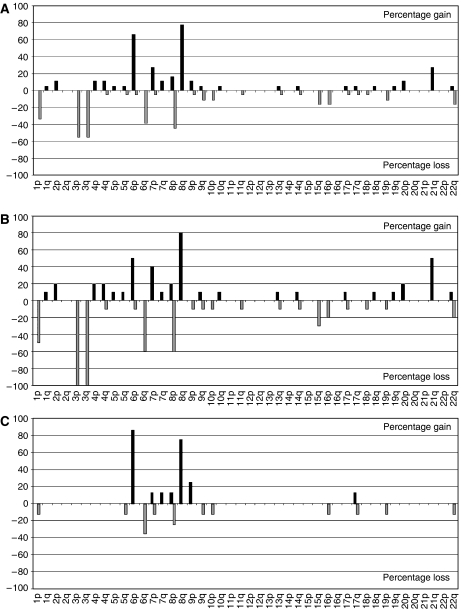
Summary of chromosomal losses and gains in (**A**) all tumours (*n*=18), (**B**) monosomy 3 (*n*=10) and (**C**) disomy 3 (*n*=8) tumours. Proportion of tumours of each type with gain and loss at the level of the chromosome arm are shown by black and grey bars, respectively.

The data presented here are based on the array CGH analysis of 18 UVMs. Figure 1 shows representative CGH profiles for chromosome 6, 8 and 21. Table 2 shows the chromosomal changes for all 18 tumours analysed, and Figure 2 shows the frequency of CNAs detected at the level of each chromosome arm. The overall frequency of alterations observed in the 18 tumours was higher than has been previously reported in metaphase CGH studies of UVM (Ghazvini *et al*, 1996; Tschentscher *et al*, 2000; Aalto *et al*, 2001; Naus *et al*, 2001). This reflects the greater sensitivity of array CGH.

Previous reports have stated that the most frequent anomaly in UVM is loss of an entire copy of chromosome 3 (Sisley *et al*, 1992; Wiltshire *et al*, 1993; White *et al*, 1998). In our analysis, however, the most common chromosomal changes identified were 8q gains (14/18; 78, 95% confidence interval: 52–94%), 6p gains (12/18; 78, 95% confidence interval: 41–87%), and monosomy 3 (10/18; 56, 95% confidence interval: 31–78%). Less common CNAs were loss of 1p (6/18; 33%), 6q (7/18; 39%) and 8p (8/18; 44%), and gain of 7p (5/18; 28%) and 21q (5/18; 28%).

Inspection of the CGH profiles of monosomy 3 and disomy 3 tumours delineated two tumour types; a division supported by hierarchical clustering of the CGH data (see Supplementary data) and published microarray expression data (Tschentscher *et al*, 2003). In all, 10 tumours had monosomy 3 and eight disomy 3. Figure 2 shows the frequency of genomic imbalance, at the chromosome arm level, stratified by chromosome 3 status. Compared to disomy 3 melanomas, monosomy 3 tumours showed more frequent loss of chromosomal material from 1p, 6q and 8p in addition to gain of 7 and 21q. Furthermore, the regions of change in disomy 3 tumours, in some cases, involved less of the affected chromosomal arms than monosomy 3 tumours (Table 2). This probably reflects increase genomic instability in monosomy 3 tumours (Myatt *et al*, 2000).

Previously published analyses of chromosome 3 in UVM have shown that partial deletions of monosomy 3 are detectable in approximately 50% of tumours and have defined two minimum regions of loss, 3p25.1–p25.2 and 3q24–q26 (Tschentscher *et al*, 2001; Parrella *et al*, 2003). All the tumours we analysed with loss of chromosome 3 material showed loss of the entire chromosome, thus we were not able to further refine either of these regions of deletion.

Monosomy 3 has been hypothesised to represent an early event in tumourigenesis, defining a bificated tumour progression pathway (Parrella *et al*, 1999; Hoglund *et al*, 2004). In our study there was an inverse relationship between monosomy 3 and gain of 6p (Fishers exact test *P*=0.06). It has been proposed that at least two cytogenetic pathways of clonal evolution exist for UVMs, one initiated with monosomy 3 and one with gain of 6p (Hoglund *et al*, 2004).

UVMs characterised by monosomy 3 are associated with a greater tumour size and a poor prognosis for survival (Prescher *et al*, 1996; Sisley *et al*, 1997; Scholes *et al*, 2003). Although our study did not permit survivorship associated with monosomy 3 to be formally assessed, it is noteworthy that six of the 10 patients with monosomy 3 tumours developed metastatic disease compared with only one of the eight patients with disomy 3 tumours (Fishers exact test *P*=0.06). In our study, excluding anomalies of chromosome 3, the frequency of CNAs were higher, albeit nonsignificantly, in the tumours with monosomy 3 than in tumours with disomy 3 (Mann–Whitney test, *P*<0.10); 7.5 CNAs (range 2–20) and 3.5 CNAs (range 1–13) respectively. Recent work on other cancers has shown prognosis is worse when high rates of chromosome instability are a feature, probably a consequence of the accumulation of induced genetic alterations (Nakamura *et al*, 2003). This might explain the better prognosis observed for UVM patients with disomy 3 tumours. In our study tumours with monosomy 3 were larger than those with disomy 3 (Mann–Whitney test, *P*<0.008; median sizes 18.4 mm (range 16.2–20.9 mm) and 13.9 mm (range 11.8–22.2 mm, respectively) and after adjusting for tumour size there was little support for a relationship between monosomy 3 status and frequency of CNAs *per se* (*P*=0.80). Although only a small number of tumours were analysed in our study this finding invites speculation that monosomy 3 may be a consequence of clonal selection during tumour progression.

To identify SORI at each region of CNA, genomic distances spanning regions of imbalance were determined separately for monosomy 3 and disomy 3 tumours, at the resolution of individual BAC clones. The data from tumours in each group were then compared and the SORI defined for each chromosome. The SORI involving at least two of the 10 monosomy 3 tumours or two of the eight disomy 3 tumours are shown in Table 3. The SORI data described here (Table 3) corroborate the previously identified regions of imbalance reported to be associated with UVMs (Aalto *et al*, 2001; Naus *et al*, 2001), specifically, 6p (6p25.1–p21.2), 6q (6q16.2–q25.3) and 8p (8p23.3–p11.23). Furthermore, our SORI data delineates a number of additional minimal regions of imbalance in monosomy 3 patients, several less than 30 Mb, as detailed in Table 3.

In addition to confirming and refining, at base pair resolution, a number of previously reported CNAs, the use of high-resolution array CGH has allowed us to accurately delineate (to within 1 Mb) a number of rarely reported chromosomal regions of abnormality in UVM. The minimum regions defined are likely to harbour genes important to the development of UVMs.

## Figures and Tables

**Table 1:** Clinico-pathological characteristics of patients analysed

**Tumour ID**	**Gender**	**Age at diagnosis (years)**	**Tumour size (mm)**	**Cell type**	**Metastatic death from UVM**	**Chromosome 3 status**
15	Male	49	18.0	Mixed	+	Monosomy
18	Male	72	18.5	Mixed	+	Monosomy
24	Male	73	17.5	Mixed	+	Monosomy
33	Male	50	12.8	Mixed	−	Disomy
40	Male	86	18.8	Mixed	+	Monosomy
41	Male	29	11.8	Mixed	−	Disomy
43	Male	67	18.4	Mixed	+	Monosomy
44	Male	72	17.9	Mixed	−a	Monosomy
53	Male	42	20.9	Spindle cell	−	Monosomy
55	Male	67	14.2	Spindle cell	+	Disomy
59	Male	57	15.8	Mixed	−	Disomy
64	Female	42	16.2	Mixed	−	Monosomy
67	Female	65	19.7	Mixed	−	Monosomy
69	Female	51	13.6	Mixed	−	Disomy
75	Male	54	22.2	Mixed	−	Disomy
76	Male	57	18.4	Mixed	+	Monosomy
79	Female	81	14.2	Spindle cell	−b	Disomy
81	Male	52	12.0	Mixed	−	Disomy

a Mortality from bronchial carcinoma.

b Mortality from noncancer related disease.

**Table 2:** Overview of genetic changes

**Tumour sample**	**Loss**	**Gain**
15	3p26.3–q27.3, 8p23.3–p11.1	8q11.1–q24.23
18	3p26.3–q27.3, 6q11.1–q25.3, 8p23.3–p11.23	2p24.3–p13.2, 5p15.32–p13.1, 5q11.2–q12.3,
		5q13.2–q35.3, 6p25.3–p11.1, **8q11.1–q24.3**, 10q22.1–q22.3, 10q23.31–q24.31, 10q26.13–q26.3
24	3p26.3–q27.3, 6q11.1–q25.3, 8p23.3–p11.1	2p25.3–q37.3, 6p25.3–p12.1, 7p22.3–q36.3, **8q11.1–q24.3**, 17p13.3–q24.3, 21q11.2–q22.3
33	7p22.3–p12.2, 8p23.3–p11.23	3p12.3–3p11.2
40	1p36.33–p34.3, 1p31.1–q21.1, 3p26.3–q27.3, 6q16.1–q25.3	**8q11.1–q24.3**, 21q21.2–q21.3
41	10p15.3–p12.33	6p25.3–p22.3, **8q12.2–q24.3**, 17q23.2–q24.1
43	1p36.33–p34.3, 3p26.3–q27.3, 6q12.2–q25.3,	4q28.3–q31.3, 6p25.2–p12.1, 7p22.1–q36.3, 8q11.2–q24.3
	8p23.3–p11.21, 10p15.3–q26.3, 11q12.3–q13.1, 16p13.12–p12.2, 19p13.3–q13.43, 22q11.1–q13.31	
44	3p26.3–q27.3, 6q16.2–q25.3, 8p23.3–p11.1	6p25.3–p21.2, 8q11.1–q24.3
53	1p36.33–q21.2, 3p26.3–q27.3, 9q21.2–q31.3,	4p16.1–p15.1, 4q22.3–q28.1, 6p25.2–q25.3, 7p22.1–q36.3
	15q11.2–q26.3	8p23.3–q24.3, 9q33.1–q34.3, 13q12.11–q33.3,
		18q22.2–q22.3, 19q13.2–q13.41, 19q13.2–q13.41, 20p12.3–q13.33, 21q21.1–q22.3, 22q11.21–q13.33
55		6p25.3–p12.1, 8q21.13–q24.3
59	6q11.1–q25.3	6p25.3–p12.1, 8q13.3–q24.3, 9p13.2–q34.3
64	1p36.33–p11.2, 3p26.3–q27.3, 4q13.3–q35.2, 15q11.2–q26.3	1q21.1–q42.3, 4p16.2–p13.1, 8p23.3–q24.3, 21q11.2–q22.3
67	1p36.33–q42.3, 3p26.3–q27.3, 6p25.3–q25.3, 13q12.11–q33.3, 14q11.2–q32.33, 15q11.1–q26.3, 16p13.3–q24.3, 17p11.2–q25.3, 18p11.32–q22.3, 22q11.1–q13.33	7p22.3–q36.3, 20p12.3–q13.33, 21q22.13–q22.3
69	6q16.3–q25.3, 8p23.3–p11.1	6p25.3–p12.1, 8q23.2–q24.3
75	1p36.22–p34.2, 5q35.3, 9q34.2–q34.3, 16p13.12–p12.2, 17q21.1–q21.31, 19p13.2–q13.43, 22q11.21–q13.31	6p25.3–q11.1, 7p21.3–q36.3, 8p23.2–q24.3
76	3p26.3–q27.3, 8p23.3–p11.1	8q11.1–q24.3
79		6p25.3–p11.1
81	6q11.1–q25.3	6p25.3–p12.1, 8q13.3–q24.3, 9p24.3–q21.3

Highly amplified regions in bold.

**Table 3:** Smallest overlapping regions of imbalance found by array CGH for patients displaying (a) monosomy 3 (*n*=10) and (b) disomy 3 (*n*=8), in addition, to the genes either over expressed or under expressed with in these regions

**Affected region**	**Smallest overlapping region of imbalance (SORI)**	**SORI incidence**	**Chromosome position in base pairs**	**Mb size**	**Approximate number of genes**	Genes reported to be differentially expressed in UVM*
**Gains**						**Upregulated**
*(a)*						
2p25.3–q37.3	2p24.3–p13.2	2/10 (20%)	16 036 517–68 981 280	52.9	180	
4p16.1–p15.1	4p16.1–p15.1	2/10 (20%)	10 782 302–34 782 661	24.0	42	
6p25.3–p11.1	6p25.1–p21.2	5/10 (50%)	5 060 091–37 828 169	32.7	380	HMGIY, HLA-DPA1
7p22.3–q36.3	7p22.1–q36.3	4/10 (40%)	7 725 571–158 345 327	150.6	862	EGFR, HOXA11 and 13, HGF, CDK6, EPO, BRAF
8p23.3–q24.3	8q11.21–q24.3	8/10 (80%)	49 397 958–146 062 919	96.7	367	RRM2B, TSPYL5, CGI-12, PRKDC, TAF2
20p12.3–q13.33	20p12.3–q13.33	2/10 (20%)	6 285 715–63 648 414	57.3	414	JAG1
21q11.2–q22.3	21q21.2–q21.3	4/10 (40%)	24 509 084–28 625 616	4.1	11	SOD1
	21q22.13–q22.3	4/10 (40%)	34 482 486–46 798 642	12.3	135	S100B
						
**Losses**						**Downregulated**
1p36.33–q21.3	1p36.33–p34.3	5/10 (50%)	1 059 869–36 259 335	35.2	399	PTP4A2, VAMP3,
	1p31.1–p21.2	4/10 (40%)	73 223 190–94 336 358	29.1	112	IL12RB2, PDE4B, PLXNB1,
3p26.3–q27.3	3p26.3–q27.3	10/10 (100%)	186 817–198 819 498	198.6	944	CHL1, H1FX, fls485, ROBO1, SETMAR, IL1RAP, NR1D2, RAF1, MBD4, CTNNB1, eIF2a, RPL24, GC20, RPL15, PIK3R4, FXR1, TKT, RAP2B, WIG1
6q11.1–q25.3	6q16.2–q25.3	6/10 (60%)	96 260 894–170 637 357	74.3	291	
8p23.3–p11.1	8p23.3–p11.23	6/10 (60%)	477 644–35 173 110	34.8	160	ATIP1
15q11.1–q26.3	15q11.2–q26.3	3/10 (30%)	20 401 349–99 832 748	79.4	354	SMAD3
16p13.12–16p12.2	16p13.12–16p12.2	2/10 (20%)	13 892 248–21 963 275	8.0	57	COX6A2, MVP
22q11.1–22q13.31	22q11.1–22q13.31	2/10 (20%)	15 615 802–44 352 473	28.8	352	TIMP3, PDGFB
						
*(b)*						
**Gains**						**Unregulated**
6p25.3–p11.1	6p25.3–p22.3	7/8 (87.5%)	90 997–19 131 250	18.2	81	HMGIY, HLA-DPA1
8q12.2–q24.3	8q23.2–q24.3	6/8 (75%)	108 867 491–146 062 919	37.2	164	TAF2
9p13.2–9q21.33	9p13.2–9q21.33	2/8 (25%)	28 840 514–79 219 038	50.4	127	
						
**Losses**						**Downregulated**
6q14.1–6q25.3	6q16.3–q25.3	3/8 (37.5%)	97 763 086–170 637 357	72.9	291	
8p23.3–p11.23	8p23.3–p11.23	2/8 (25%)	477 644–35 173 110	34.8	160	ATIP1

* Data extracted from Dunne *et al*, 1998; Hendrix *et al*, 1998; Hurks *et al*, 2000; Onken *et al*, 2004; Radosevich *et al*, 2004; Tschentscher *et al*, 2003; van der Velden *et al*, 2003; Van Ginkel *et al*, 1998; Zuidervaart *et al*, 2003.

## References

[bib1] Aalto Y, Eriksson L, Seregard S, Larsson O, Knuutila S (2001) Concomitant loss of chromosome 3 and whole arm losses and gains of chromosome 1, 6, or 8 in metastasizing primary uveal melanoma. Invest Ophthalmol Vis Sci 42: 313–317 11157859

[bib2] Beheshti B, Braude I, Marrano P, Thorner P, Zielenska M, Squire JA (2003) Chromosomal localization of DNA amplifications in neuroblastoma tumors using cDNA microarray comparative genomic hybridization. Neoplasia 5: 53–62 12659670 10.1016/s1476-5586(03)80017-9 PMC1502121

[bib3] Bergman L, Seregard S, Nilsson B, Lundell G, Ringborg U, Ragnarsson-Olding B (2003) Uveal melanoma survival in Sweden from 1960–1998. Invest Ophthalmol Vis Sci 44: 2579–2583 10.1167/iovs.03-0081 12882771

[bib4] Douglas EJ, Fiegler H, Rowan A, Halford S, Bicknell DC, Bodmer W, Tomlinson IP, Carter NP (2004) Array comparative genomic hybridization analysis of colorectal cancer cell lines and primary carcinomas. Cancer Res 64: 4817–4825 15256451 10.1158/0008-5472.CAN-04-0328

[bib5] Dunne BM, McNamara M, Clynes M, Shering SG, Larkin AM, Moran E, Barnes C, Kennedy SM (1998) MDR1 expression is associated with adverse survival in melanoma of the uveal tract. Hum Pathol 29: 594–598 9635679 10.1016/s0046-8177(98)80008-7

[bib6] Fiegler H, Carr P, Douglas EJ, Burford DC, Hunt S, Scott CE, Smith J, Vetrie D, Gorman P, Tomlinson IP, Carter NP (2003) DNA microarrays for comparative genomic hybridization based on DOP-PCR amplification of BAC and PAC clones. Genes Chromosomes Cancer 36: 361–374 12619160 10.1002/gcc.10155

[bib7] Ghazvini S, Char DH, Kroll S, Waldman FM, Pinkel D (1996) Comparative genomic hybridization analysis of archival formalin-fixed paraffin-embedded uveal melanomas. Cancer Genet Cytogenet 90: 95–101 8830715 10.1016/s0165-4608(96)00076-3

[bib8] Hendrix MJ, Seftor EA, Seftor RE, Kirschmann DA, Gardner LM, Boldt HC, Meyer M, Pe'er J, Folberg R (1998) Regulation of uveal melanoma interconverted phenotype by hepatocyte growth factor/scatter factor (HGF/SF). Am J Pathol 152: 855–863 9546344 PMC1858259

[bib9] Hoglund M, Gisselsson D, Hansen GB, White VA, Sall T, Mitelman F, Horsman D (2004) Dissecting karyotypic patterns in malignant melanomas: temporal clustering of losses and gains in melanoma karyotypic evolution. Int J Cancer 108: 57–65 14618616 10.1002/ijc.11558

[bib10] Hurks HM, Metzelaar-Blok JA, Barthen ER, Zwinderman AH, De Wolff-Rouendaal D, Keunen JE, Jager MJ (2000) Expression of epidermal growth factor receptor: risk factor in uveal melanoma. Invest Ophthalmol Vis Sci 41: 2023–2027 10892838

[bib11] McLean IW, Saraiva VS, Burnier Jr MN (2004) Pathological and prognostic of uveal melanomas. Can J Ophthalmol 39: 343–350 15327098 10.1016/s0008-4182(04)80004-8

[bib12] Myatt N, Aristodemou P, Neale MH, Foss AJ, Hungerford JL, Bhattacharya S, A Cree I (2000) Abnormalities of the transforming growth factor-beta pathway in ocular melanoma. J Pathol 192: 511–518 11113869 10.1002/1096-9896(2000)9999:9999<::AID-PATH778>3.0.CO;2-B

[bib13] Nakamura H, Saji H, Idiris A, Kawasaki N, Hosaka M, Ogata A, Saijo T, Kato H (2003) Chromosomal instability detected by fluorescence *in situ* hybridization in surgical specimens of non-small cell lung cancer is associated with poor survival. Clin Cancer Res 9: 2294–2299 12796398

[bib14] Naus NC, van Drunen E, de Klein A, Luyten GP, Paridaens DA, Alers JC, Ksander BR, Beverloo HB, Slater RM (2001) Characterization of complex chromosomal abnormalities in uveal melanoma by fluorescence *in situ* hybridization, spectral karyotyping, and comparative genomic hybridization. Genes Chromosomes Cancer 30: 267–273 11170284

[bib15] Onken MD, Worley LA, Ehlers JP, Harbour JW (2004) Gene expression profiling in uveal melanoma reveals two molecular classes and predicts metastatic death. Cancer Res 64: 7205–7209 15492234 10.1158/0008-5472.CAN-04-1750 PMC5407684

[bib16] Parrella P, Fazio VM, Gallo AP, Sidransky D, Merbs SL (2003) Fine mapping of chromosome 3 in uveal melanoma: identification of a minimal region of deletion on chromosomal arm 3p25.1–p25.2. Cancer Res 63: 8507–8510 14679017

[bib17] Parrella P, Sidransky D, Merbs SL (1999) Allelotype of posterior uveal melanoma: implications for a bifurcated tumor progression pathway. Cancer Res 59: 3032–3037 10397238

[bib18] Prescher G, Bornfeld N, Hirche H, Horsthemke B, Jockel KH, Becher R (1996) Prognostic implications of monosomy 3 in uveal melanoma. Lancet 347: 1222–1225 8622452 10.1016/s0140-6736(96)90736-9

[bib19] Radosevich M, Song Z, Gorga JC, Ksander B, Ono SJ (2004) Epigenetic silencing of the CIITA gene and posttranscriptional regulation of class II MHC genes in ocular melanoma cells. Invest Ophthalmol Vis Sci 45: 3185–3195 15326139 10.1167/iovs.04-0111

[bib20] Scholes AG, Damato BE, Nunn J, Hiscott P, Grierson I, Field JK (2003) Monosomy 3 in uveal melanoma: correlation with clinical and histologic predictors of survival. Invest Ophthalmol Vis Sci 44: 1008–1011 12601021 10.1167/iovs.02-0159

[bib21] Sisley K, Cottam DW, Rennie IG, Parsons MA, Potter AM, Potter CW, Rees RC (1992) Non-random abnormalities of chromosomes 3, 6, and 8 associated with posterior uveal melanoma. Genes Chromosomes Cancer 5: 197–200 1384670 10.1002/gcc.2870050304

[bib22] Sisley K, Rennie IG, Parsons MA, Jacques R, Hammond DW, Bell SM, Potter AM, Rees RC (1997) Abnormalities of chromosomes 3 and 8 in posterior uveal melanoma correlate with prognosis. Genes Chromosomes Cancer 19: 22–28 9135991 10.1002/(sici)1098-2264(199705)19:1<22::aid-gcc4>3.0.co;2-2

[bib23] Stang A, Parkin DM, Ferlay J, Jockel KH (2005) International uveal melanoma incidence trends in view of a decreasing proportion of morphological verification. Int J Cancer 114: 114–123 15523698 10.1002/ijc.20690

[bib24] Tschentscher F, Husing J, Holter T, Kruse E, Dresen IG, Jockel KH, Anastassiou G, Schilling H, Bornfeld N, Horsthemke B, Lohmann DR, Zeschnigk M (2003) Tumor classification based on gene expression profiling shows that uveal melanomas with and without monosomy 3 represent two distinct entities. Cancer Res 63: 2578–2584 12750282

[bib25] Tschentscher F, Prescher G, Horsman DE, White VA, Rieder H, Anastassiou G, Schilling H, Bornfeld N, Bartz-Schmidt KU, Horsthemke B, Lohmann DR, Zeschnigk M (2001) Partial deletions of the long and short arm of chromosome 3 point to two tumor suppressor genes in uveal melanoma. Cancer Res 61: 3439–3442 11309305

[bib26] Tschentscher F, Prescher G, Zeschnigk M, Horsthemke B, Lohmann DR (2000) Identification of chromosomes 3, 6, and 8 aberrations in uveal melanoma by microsatellite analysis in comparison to comparative genomic hybridization. Cancer Genet Cytogenet 122: 13–17 11104026 10.1016/s0165-4608(00)00266-1

[bib27] van der Velden PA, Zuidervaart W, Hurks MH, Pavey S, Ksander BR, Krijgsman E, Frants RR, Tensen CP, Willemze R, Jager MJ, Gruis NA (2003) Expression profiling reveals that methylation of TIMP3 is involved in uveal melanoma development. Int J Cancer 106: 472–479 12845640 10.1002/ijc.11262

[bib28] Van Ginkel PR, Gee RL, Walker TM, Hu DN, Heizmann CW, Polans AS (1998) The identification and differential expression of calcium-binding proteins associated with ocular melanoma. Biochim Biophys Acta 1448: 290–297 9920419 10.1016/s0167-4889(98)00133-5

[bib29] White VA, McNeil BK, Horsman DE (1998) Acquired homozygosity (isodisomy) of chromosome 3 in uveal melanoma. Cancer Genet Cytogenet 102: 40–45 9530338 10.1016/s0165-4608(97)00290-2

[bib30] Wiltshire RN, Elner VM, Dennis T, Vine AK, Trent JM (1993) Cytogenetic analysis of posterior uveal melanoma. Cancer Genet Cytogenet 66: 47–53 8467475 10.1016/0165-4608(93)90148-f

[bib31] Zuidervaart W, van der Velden PA, Hurks MH, van Nieuwpoort FA, Out-Luiting CJ, Singh AD, Frants RR, Jager MJ, Gruis NA (2003) Gene expression profiling identifies tumour markers potentially playing a role in uveal melanoma development. Br J Cancer 89: 1914–1919 14612903 10.1038/sj.bjc.6601374 PMC2394439

