# Supplementary figures and images for: Microarray comparative genomic hybridisation analysis of intraocular uveal melanomas identifies distinctive imbalances associated with loss of chromosome 3
Source: Br J Cancer. 2005 Oct 25;93(10):1191–6. doi: 10.1038/sj.bjc.6602834 (PMC2361503; doi:10.1038/sj.bjc.6602834)

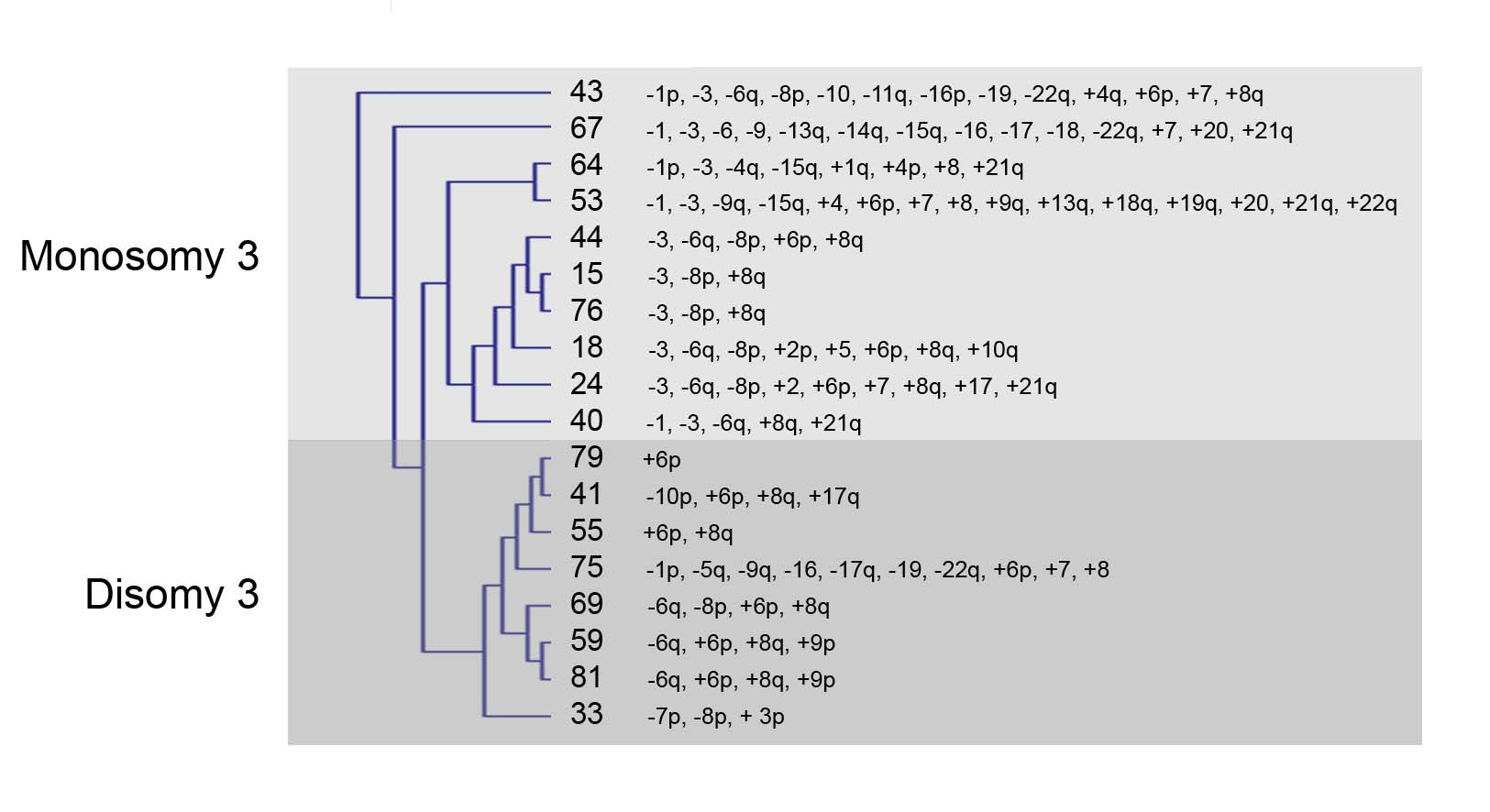

Supplement: Supplementary Data [file 93-6602834x1.jpg]
